# Parahippocampus hypertrophy drives gray matter morphological alterations in migraine patients without aura

**DOI:** 10.1186/s10194-023-01588-z

**Published:** 2023-05-17

**Authors:** Tao Yin, Lei Lan, Zilei Tian, Zhengjie Li, Mailan Liu, Yujie Gao, Fanrong Liang, Fang Zeng

**Affiliations:** 1 Acupuncture and Tuina School, Chengdu University of Traditional Chinese Medicine, Chengdu, 610075 Sichuan China; 2 Acupuncture and Brain Science Research Center, Chengdu University of Traditional Chinese Medicine, Chengdu, 610075 Sichuan China; 3 College of Acupuncture & Moxibustion and Tuina, Hunan University of Chinese Medicine, Changsha, 410208 Hunan China; 4 Traditional Chinese Medicine School, Ningxia Medical University, Yinchuan, 750004 Ningxia China; 5 Key Laboratory of Sichuan Province for Acupuncture and Chronobiology, Chengdu, 610075 Sichuan China

**Keywords:** Migraine, Migraine without aura, Gray matter morphology, Structural covariance network, Causal structural covariance network

## Abstract

**Background:**

The aberrance of gray matter morphology in migraineurs has been widely investigated. However, it remains largely unknown whether there are illness duration-related hierarchical changes in the gray matter structure.

**Methods:**

A total of 86 migraine without aura (MwoA) patients and 73 healthy controls were included. The Voxel-Based Morphometry approach was utilized to compare the gray matter volume (GMV) differences between MwoA patients and healthy controls. The Structural Covariance Network analysis was conducted to quantify the cross-regional synchronous alterations of gray matter structure in MwoA patients. The Causal Structural Covariance Network analysis was performed to describe the progressive and hierarchical changes in the gray matter network of patients in the pathological progression of migraine.

**Results:**

MwoA patients had duration-stage related GMV hypertrophy in the left parahippocampus, as well as synergistic GMV aberrance in the parahippocampus and the medial inferior temporal gyrus and cerebellum. Moreover, the GMV alteration of the parahippocampus, and the surrounding hippocampus, amygdala, and bilateral anterior cerebellum, preceded and causally influenced the morphological changes of lateral parietal-temporal-occipital gyrus, as well as the motor cortex and prefrontal gyrus with the increasing illness duration in MwoA patients.

**Conclusion:**

The current study indicated that gray matter structural alterations in the medial inferior temporal gyrus, especially the parahippocampus, is a critical pathological characteristic in MwoA patients, which drives the gray matter structure alteration of other regions. These findings provide further evidence for understanding the progressive gray matter morphological changes in migraine and may facilitate the development of neuromodulation therapies targeting this procession.

## Introduction

Migraine is a chronic paroxysmal neurological disorder manifested as recurring episodes of headache, often accompanied by various neurological and systematic symptoms [1]. Depending on whether the attack is preceded or accompanied by focal symptoms such as visual disturbances, tingling and numbness in the face, and confusion, the migraine is classified as migraine with and without aura [2]. As estimated in the latest epidemiological survey, the global prevalence of migraine reached up to 14–15% [3], with over 70% of cases being migraine without aura (MwoA) [4]. Its widespread prevalence, and associated disability, impose a range of negative and substantial effects on individuals and society [4, 5].

Despite the tremendous burdens of migraine, knowledge regarding its pathophysiology remains very limited, which makes it especially challenging to the objective diagnosis and precise treatment. For instance, the current diagnosis of migraine is based on the patient's medical history and nonspecific physical examination, which relies heavily on the physician's experience [2, 6]. To address this issue, there has been a growing consensus to identify measurable biomarkers associated with the migraine process so as to detect individuals at risk for the disorder and facilitate the development of novel interventions [7]. Magnetic resonance imaging (MRI) has provided critical insights into migraine pathogenesis and set the stage for the development of imaging-based biomarkers [8, 9]. Accumulating structural MRI studies have been performed in migraine patients and reported morphological alterations of the brain at both the regional and network levels [10]. Alterations of gray matter structure in the temporal gyrus, prefrontal gyrus, subcortex region, etc., have been reliably detected and replicated across cohorts worldwide [11–14]. Furthermore, a proof-of-concept study utilized the structural brain MRI measure to develop classifiers that accurately differentiated chronic migraineurs from episodic migraineurs and healthy subjects [15]. However, these significant findings were achieved based on the cross-sectional data and could hardly reflect the temporal and causal characteristics of morphological alterations in the gray matter of migraine patients. It remains largely unknown whether there are illness duration-related hierarchical changes in the gray matter structure (i.e., whether the disruption in one region is secondary to that in another) of migraine patients.

Granger causality (GC) analysis is a widely used technique for time-series data [16]. It enables the tracking of information flow within the brain by detecting whether neural activity in one region precedes and allows for the prediction of activity in another region [17]. Generally, GC analysis is applied to 4D functional MRI instead of structural MRI data since it lacks necessary time-series information. Whereas, if morphological data are ranked according to the information of disease progression and duration and are given temporal information, GC analysis can be performed on these sequential data to assess the cross-region causal relationship of structural brain alterations. This analysis strategy was called Causal Structural Covariance Network (CaSCN) [18]. Here, we aimed to utilize CaSCN to describe the illness duration-related hierarchical changes in the gray matter structure of migraineurs, thereby providing a progressive perspective for understanding brain structural alterations in migraineurs.

Since MwoA is the most common type of migraine [4], the present study focused on the progressive alterations in the gray matter structure of MwoA patients. First, the Voxel-Based Morphometry (VBM) approach was utilized to compare the gray matter volume (GMV) differences between MwoA patients and healthy controls (HCs), followed by the subgroup comparison of patients at different stages of illness duration. The purpose of these comparative analyses was to identify the key regions with significant morphological alterations associated with different stages of MwoA. Subsequently, Structural Covariance Network (SCN) analysis was performed based on the identified key regions to quantify the cross-regional synchronous alterations of gray matter structure in the pathological process of migraine. Finally, the CaSCN method was utilized to describe the progressive changes in the gray matter structure in MwoA patients as the duration increased. We hypothesized that the GMV of MwoA patients would gradually change with increasing illness duration and that these changes may be causally and hierarchically correlated.

## Materials and methods

### Participants

Eighty-six MWoA patients and seventy-six HCs were enrolled. The patients were recruited from the outpatients of the Third Affiliated Hospital of Chengdu University of Traditional Chinese Medicine and were diagnosed by a neurologist according to the 2nd Edition International Classification of Headache Disorders for Migraine Without Aura [19]. Patients were included if they fulfilled the following criteria: 1) between 17 to 45 years old, 2) right-handed, 3) suffered from migraine symptoms for at least six months, 4) had at least one attack per month in the past three months, 5) were not taking any analgesics treatment for migraine in the last three months. Patients were excluded if they: 1) were diagnosed with a secondary headache caused by traumatic brain injury, hypertension, or any other organic reasons, 2) comorbid with any other psychoneurological disorders or had any severe primary illnesses, 3) had any other chronic pain conditions, 4) had contraindication of MRI scanning, e.g., claustrophobia.

Seventy-six right-handed, gender- and age-matched HCs were recruited by advertisements from the campus of Chengdu University of Traditional Chinese Medicine. These participants were free from any chronic pain disorder or any other organic or functional diseases.

Both MwoA patients and HCs underwent comprehensive history taking, physical examination, and routine laboratory examinations. Subjects with abnormal test results were excluded.

### Symptoms evaluation

The included MwoA patients were required to record headache diaries to evaluate the clinical symptoms in the one-month observation period. The headache diaries contained two dimensions, the day with migraine attacks and the intensity of the headache that was evaluated with the 0–10 Visual Analogue Scale (VAS) score. Subsequently, the monthly migraine days were calculated by summing the number of days with migraine attacks in the observation period, while the headache intensity was assessed by the averaged VAS score of each migraine attack. Additionally, the Self-rating Anxiety Scale [20] and Self-Rating depressive Scale [21] were utilized to evaluate the emotional conditions of MwoA patients.

The demographic characteristics of MwoA patients and HCs were compared via SPSS 20.0 software (SPSS Inc. USA) with a significance threshold of *p* < 0.05 (two-tailed). The clinical symptoms of patients were described with mean ± standard deviation.

### MRI data acquisition

MRI data of patients and HCs were acquired with the same 3.0 T MRI scanner (Siemens AG, Germany) at West China Hospital of Sichuan University. The high-resolution 3-Dimensional T1-Weighted Imaging was obtained with the axial fast spoiled gradient recalled sequence. The scanning parameters were as follows: repetition time/echo time = 1900/2.26 ms, slice thickness = 1 mm, the field of view = 256 × 256 mm^2^, and matrix size = 256 × 256. MwoA patients were migraine-free for at least 72 h prior to the MRI scan.

### Data preprocessing

The T1 image data was preprocessed with SPM12 (http://www.fil.ion.ucl.ac.uk/spm) and CAT12 (http://www.neuro.uni-jena.de/cat) toolboxes. Specifically, the data preprocessing procedure contained four steps: 1) normalizing T1 images to the standard Montreal Neurological Institute (MNI) space and segmenting images into the gray matter, white matter, and cerebrospinal fluid using DARTEL algorithm [22]; 2) estimating sample homogeneity and visually checking the images whose heterogeneity were greater than mean ± two standard deviations; 3) calculating the total intracranial volume (TIV) for each subject based on the unsmoothed images; 4) smoothing spatial images with an 8 mm Gaussian kernel of full-width at half maximum following the operation manual recommended.

### VBM analysis

The VBM analysis was utilized to quantify the GMV of the brain in each subject. The whole-brain GMV difference between MwoA patients and HCs was compared with the two-sample *t-test*, using age, gender, and TIV as covariates. The significance threshold was set to voxel *p* < 0.001 and cluster *p* < 0.05, Gaussian Random Field (GRF) correction.

To map the progressive patterns of GMV alteration in MwoA patients, we utilized stage-specific procedures [18, 23] to divide patients and make a subgroup analysis. Given the distribution of illness duration and the corresponding number of patients, these patients were categorized into two subgroups (Stage 1: the short duration, 6 ≤ illness duration < 60 months; Stage 2: the long duration, illness duration ≥ 60 months). GMV of patients in these two subgroups was compared to those of HCs using the two-sample *t-tests*, respectively. As well age, gender, and TIV were regressed as covariates in these analyses. The significance thresholds for subgroup comparisons were set at voxel *p* < 0.001 and cluster *p* < 0.05, GRF correction, which was consistent with the between-group comparison of all patients and HCs [24].

Subsequently, we extracted the average GMV from each group of subjects (patients with short duration, patients with long duration, and HCs) in the regions where GMV was altered in MwoA patients and then compared them with the *analysis of covariance* in SPSS 20.0, followed by *post-hoc* analysis between any two subgroups. The significance threshold was set at *p* < 0.05, Bonferroni correction.

To further evaluate the associations between GMV alterations and illness duration as well as symptom severity in MwoA patients, we conducted the partial correlation analysis between GMV of these altered regions and illness duration and clinical symptoms, with age, gender, and TIV as covariates. The significant threshold was set to *p* < 0.05, Bonferroni correction.

### SCN analysis

In order to investigate the cross-regional synchronization of GMV alterations in MwoA patients, we conducted the SCN analysis seeded with regions that showed significant differences in GMV between MwoA patients and HCs. Specifically, the averaged GMV of these regions was first extracted from each patient and regarded as the regressor. Then, a multi-regression model-based linear-interaction analysis was conducted in DPARSF 4.5 [25] (http://rfmri.org/DPARSF) to detect the SCN alterations in MwoA patients relative to the HCs. Age, gender, and TIV were modeled as covariates in the multi-regression analyses. Similar to the recent study [26], The statistical threshold was set to *p* < 0.05, with false discovery rate (FDR) correction.

### CaSCN analysis

To investigate whether there is a causal relationship between GMV alterations in different brain regions over time in MwoA patients (i.e., whether the damage in one region precedes that in another), the seed-based CaSCN analysis was introduced here. As shown in recent studies [23, 26, 27], CaSCN had the ability to assess the cross-region causal relationship of structural brain alterations and describe the dynamic and gradual perspective of brain structural alterations with duration increase. In detail, the GMV data of all patients were first sequenced according to the illness duration from low to high, and thereby granted “time‐series” information to the cross‐sectional data for describing the progressive property of patients. Subsequently, GC analysis was applied to this pseudo-time-series data to construct CaSCN. Similar to the SCN analysis, the seed region of CaSCN was selected from the results of the GMV comparison between MwoA patients and HCs mentioned previously. The averaged GMV values within the seed were extracted from the sequenced morphological data and constituted a pseudo time series. Then, the seed-to-voxel signed‐path coefficient GC analysis was performed using REST 1.8 (http://www.restfmri.net), [28] with age, gender, and TIV as covariates. Considered that the key assumption of GC analysis is the stationarity of data [29], *Kwiatkowski-Phillips-Schmidt-Shin* test was performed prior to the GC analysis, to ensure the stationarity of the pseudo time series of each voxel. As defined [30], if the current value of time course “Y” could be more accurately estimated by the combination of the past value of time courses “X” and “Y” than the past value of “Y” alone, “X” has Granger causal influence on Y. Accordingly, the CaSCN, by applying GC analysis to the pseudo‐time‐series morphometric data, could estimate the causal effects of morphometric alteration of a region on the others. A positive GC value indicated that the same GMV changes in the regions lagged behind the seed alteration, while a negative GC value denoted that the region with an opposite change lagged behind the seed alteration. To present the GC values with statistical parameters, the original GC map was transformed to a *z-score* map, and then the final results were reported under the threshold of *p* < 0.05, FDR correction, with a combination of *z* > 2.3 and |GC value|> 0.16 [18].

To further investigate the bidirectional causal effects among the regions obtained from seed-based CaSCN analysis, we also performed the region-of-interest (ROI) to ROI GC analysis. The ROIs were defined as 8 mm spheres centered on the peak coordinates of these regions. The signed-path coefficient GC analysis was performed to generate an ROI-wise causal network that characterized causal relationships among ROIs. To keep consistency with the voxel-wise CaSCN analysis, the same threshold was set at |GC value|> 0.16. Finally, based on the Brain Connectivity toolbox (https://www.nitrc.org/projects/bct/), the binary out-degree and in-degree values of each ROI were computed separately to identify the causal target or causal source levels of ROIs. Specifically, the in-degree value of an ROI represented the sum of the number of paths projected to the ROI, while the out-degree value of a node referred to the sum of the number of paths projecting to other nodes. The node degree was calculated with the value of out-degree + in-degree.

## Results

Since 3 HCs were excluded for the motion artifacts of the T1 image, a total of 86 MwoA and 73 HCs were included in the data analysis. The demographic characteristics of participants and the clinical symptoms of the patients are shown in Table 1. There were no statistical differences in age, gender, body mass index, and TIV between MwoA patients and HCs.

**Table 1:** Demographic characteristics and clinical symptoms of MWoA patients and HCs

	**MwoA**	**HCs**	**Statistics**	***p*-value**
Gender (Male/Female)	18/68	23/50	χ^2^ = 2.308	*p* = 0.129
Age (Year)	21.93 ± 2.25	21.62 ± 0.97	*t* = 1.036	*p* = 0.302
BMI	20.61 ± 2.40	20.83 ± 1.62	*t* = -0.693	*p* = 0.489
TIV	1422.35 ± 119.29	1439.10 ± 135.95	*t* = -0.827	*p* = 0.409
Duration (Month)	65.06 ± 35.23	/	/	/
Monthly migraine days	5.87 ± 5.21	/	/	/
Headache intensity	5.81 ± 1.80	/	/	/
SAS	45.19 ± 8.74	/	/	/
SDS	45.22 ± 10.47	/	/	/
Concomitant symptoms (Yes/No)				
Photophobia	53/34			
Phonophobia	57/30			
Nausea	48/39			
Vomiting	14/73			

*Abbreviations****:*** *MwoA* Migraine without aura, *HCs* Healthy controls, *BMI* Body mass index, *TIV* Total intracranial volume, *SAS* Zung self-rating anxiety scale, *SDS* Zung self-rating depression scale

### Overall and stage-specific GMV alterations

**Fig. 1:**
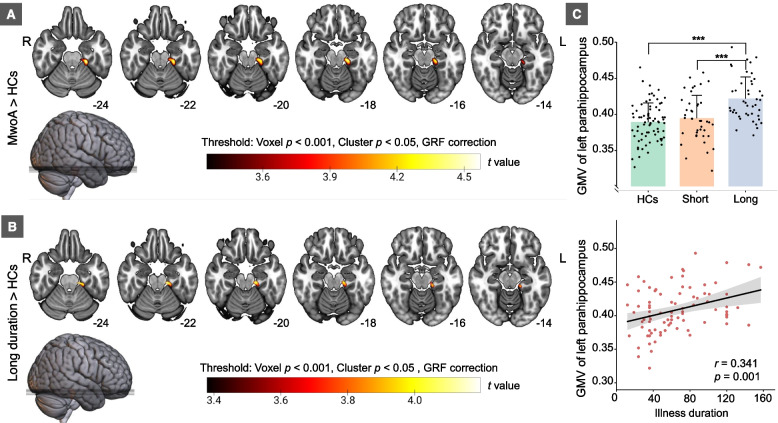
Alterations of GMV in MwoA patients. **A** indicates differences in GMV between MwoA patients and HCs. **B** shows differences in GMV between MwoA patients with long duration and HCs. **C** is the group difference of GMV of the cluster in Fig. 1A among three subgroups and the correlation of GMV and illness duration in MwoA patients. *Abbreviation****:*** *MwoA* migraine without aura, *HCs* healthy controls, *GMV* gray matter volume,* L* left, *R* right,* GRF* Gaussian Random Field

Compared to HCs, MwoA patients manifested increased GMV in the left parahippocampus (PHG.L) (Peak *t* = 4.606, Peak MNI coordinate: X = -24, Y = -30, Z = -24, cluster size = 533) under the threshold of voxel *p* < 0.001, cluster *p* < 0.05, GRF correction (Fig. 1A). Similarly, MwoA patients with long duration (*n* = 44) also showed GMV hypertrophy in the PHG.L (Peak *t* = 4.264, Peak MNI coordinate: X = -15, Y = -27, Z = -24, cluster size = 430) than HCs under the same threshold (Fig. 1B). No significant difference of whole-brain GMV was detected between MwoA patients with short duration (*n* = 42) and HCs.

There were significant differences in GMV in the PHG.L among the three subgroups (*F* = 14.903, *p* < 0.001). Specifically, patients with long duration had higher GMV than HCs and patients with short duration (long duration Vs. HCs, *t* = 6.058, *p* < 0.001; long duration Vs. short duration, *t* = 4.044, *p* < 0.001), while no significant difference of GMV between HCs and patients with short duration was found (*t* = 0.995, *p* = 0.322) (Fig. 1C).

The result of correlation analysis demonstrated a significantly positive correlation between GMV of the PHG.L and disease duration (*r* = 0.341, *p* = 0.001) (Fig. 1C), instead of with the monthly migraine days (*r* = -0.195, *p* = 0.071) or headache intensity (*r* = -0.084, *p* = 0.445) in MwoA patients.

### Synchronous GMV alterations with the PHG.L

**Fig. 2:**
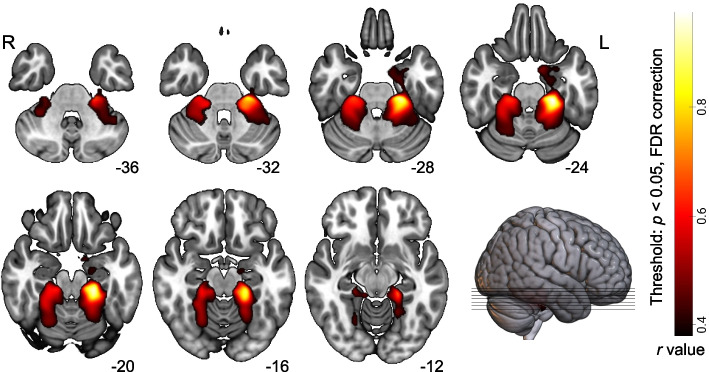
Synchronous GMV alterations along with the PHG.L in MwoA patients. *Abbreviation:* *L* left, *R* right, *FDR* false discovery rate

The RIO-based SCN analysis demonstrated a synergistic GMV increase between the PHG.L and the bilateral medial inferior temporal gyrus (including the hippocampus, parahippocampus, and fusiform gyrus) and the bilateral anterior cerebellum (including the culmen, cerebellar regions 4, 5, and 6). Specifically, two clusters survived under the threshold of *p*_*FDR*_ < 0.05. The peak coordinate of cluster 1 is located at the PHG.L (Peak MNI coordinate: X = 24, Y = -29, Z = -29; cluster size = 6828). The peak coordinate of cluster 2 is located at the right superior cerebellum (Peak MNI coordinate: X = -24, Y = -30, Z = -24; cluster size = 4802) (Fig. 2).

### Causal effects of GMV alteration in the PHG.L

**Fig. 3:**
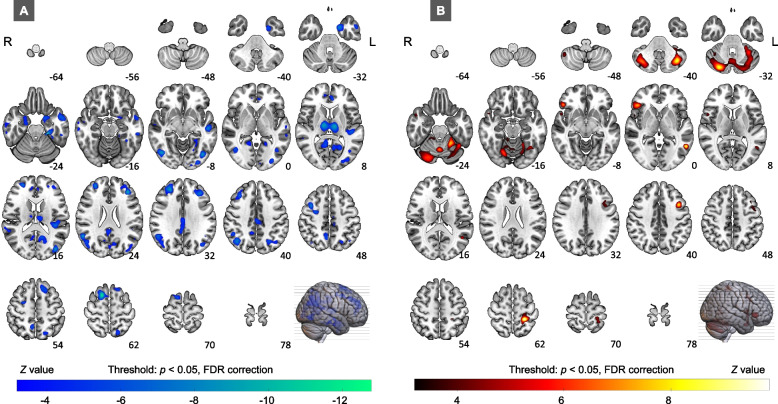
Causal effects of GMV alteration in the PHG.L. **A** illustrates the regions where the PHG.L had negative GC effects on. **B** shows the regions where the PHG.L had positive GC effects on. *Abbreviation:* *L* left, *R* right, *FDR* false discovery rate

The results of ROI-based CaSCN demonstrated negative GC from the PHG.L to the right supplementary motor area (SMA), left middle and superior frontal gyrus, left middle temporal gyrus (posterior and anterior part), and bilateral amygdala/anterior parahippocampus (AMY/aPHG), thalamus, inferior occipital gyrus, angular gyrus, precuneus, and anterior cingulate gyrus (Fig. 3A, Table 2), as well as positive GC from the PHG.L to the bilateral cerebellum, left precentral gyrus, left postcentral gyrus, right inferior orbitofrontal gyrus (IOFG), right temporal pole, and left middle temporal gyrus (temporooccipital part) (Fig. 3B, Table 2) under the threshold of *p* < 0.05, FDR correction.

**Table 2:** Causal effects of GMV alteration in the PHG.L

Regions	Abbreviations	MNI coordinates (x, y, z)	*z* value	Cluster size
R Supplementary motor area	SMA.R	15, 11, 63	-11.6	3858
L Middle frontal gyrus	MFG.L	-38, 36, 26	-9.74	1507
L Inferior occipital gyrus/angular gyrus	IOG/ANG.L	-41, -74, -6	-9.48	3900
R Angular gyrus	ANG.R	56, -56, 26	-9.21	2723
Bi Thalamus	THA.Bi	-11, -17, 11	-9.18	2528
R Inferior occipital gyrus	IOG.R	44, -71, -8	-8.5	917
L Superior frontal gyrus	SFG.L	-17, 24, 59	-8.07	742
L Amygdala/anterior parahippocampus	AMY/aPHG.L	-20, 0, -30	-8.06	1460
L Middle temporal gyrus (posterior part)	pMTG.L	-53, -21, -9	-7.86	1121
L Rolandic operculum	ROL.L	-41, -33, 15	-6.86	1319
L Precuneus	PCUN.L	-11, -57, 14	-6.8	1895
L Middle temporal gyrus (anterior part)	aMTG.L	-57, 0, -23	-6.56	1013
R Precuneus	PCUN.R	8, -54, 53	-6.18	3579
Bi Anterior cingulate gyrus	ACG.Bi	-5, 41, 8	-5.62	709
R Inferior temporal gyrus	ITG.R	63, -23, -18	-5.49	681
R Amygdala/anterior parahippocampus	AMY/aPHG.R	21, -9, -24	-5	513
R Temporal pole	TPO.R	48, 8, -29	6.14	640
L Precentral gyrus	PreCG.L	-41, 11, 41	8.18	1146
Bi Cerebellum	CRB.Bi	-38, -66, -41	8.68	13,398
L Middle temporal gyrus (temporooccipital part)	tMTG.L	-56, -57, 2	9.2	586
R Inferior orbitofrontal gyrus	IOFG.R	47, 30, -5	9.66	821
L Postcentral gyrus	PostCG.L	-24, -39, 61.5	9.84	1198

*Abbreviation:* *L* Left, *R* Right, *Bi* Bilateral, *MNI* Montreal Neurological Institute

### Bidirectional causal effects in the ROI-wise CaSCN

**Fig. 4:**
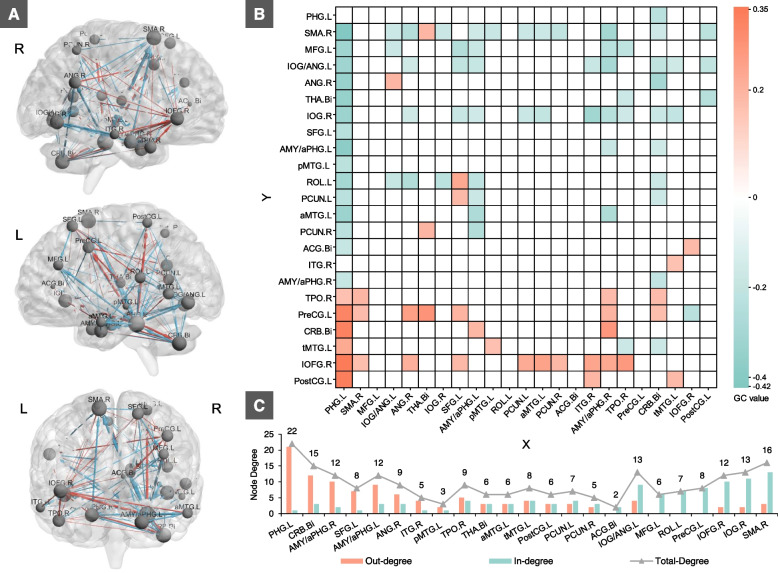
Causal connectivity among the ROI-wise CaSCN. **A** and **B** characterize the ROI-wise CaSCN, which are generated in the bidirectional signed-path coefficient GC analysis with the 3D brain networks and asymmetric matrix, respectively. **C** presents the node degree value of each ROI. *Abbreviations:* *L* left, *R* right, *GC* Granger causality, abbreviations for brain regions are listed in Table 2

As shown in Fig. 4, there is a complicated causal connectivity among these 23 ROIs. Specifically, the links between the PHG.L to right SMA, left AMY/aPHG, and bilateral thalamus were the edges with the highest negative GC value, while the links between PHG.L to IOFG.R, left postcentral gyrus, and bilateral cerebellum were the edges with highest positive GC value (Fig. 4A, B). The PHG.L, cerebellum, and AMY/aPHG were the highest out-degree nodes, projecting mainly to the other nodes, while the right SMA, left precentral gyrus, and right IOFG were the highest in-degree nodes, receiving causal effects from others. The right temporal pole, left middle temporal gyrus, bilateral thalamus, left postcentral gyrus, and bilateral precuneus were the transition nodes, showing balanced in-degree and out-degree (Fig. 4C).

## Discussion

**Fig. 5:**
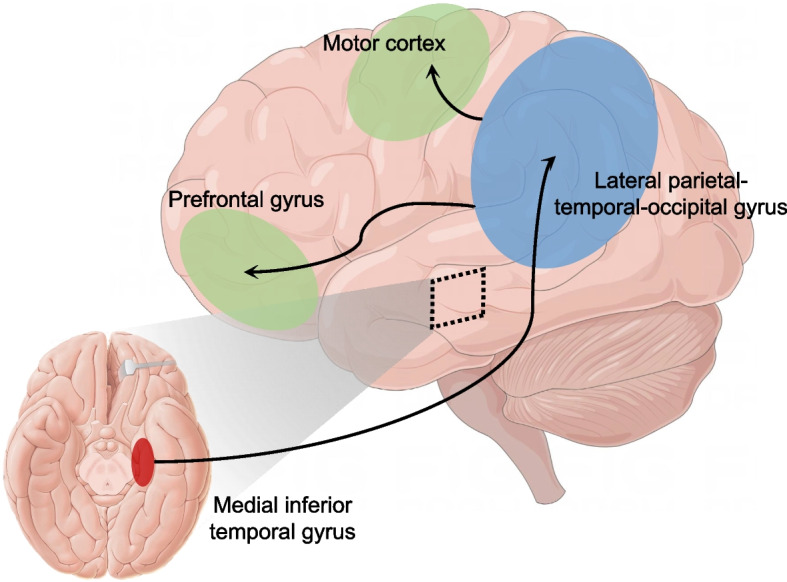
The pattern of GMV progressive alteration in MwoA patients

Adopting VBM, SCN, and CaSCN methods, the current study found that MwoA patients had duration-stage related GMV hypertrophy in the PHG.L, as well as synergistic GMV aberrance in the PHG.L and the medial inferior temporal gyrus and cerebellum. Moreover, the GMV alteration of the PHG.L, and the surrounding hippocampus, amygdala, and bilateral anterior cerebellum, preceded the morphological changes of others and represented the initiation of structural alterations in MwoA patients. These findings were consistent with our hypothesis that gray matter structure in MwoA patients showed causal and hierarchical alterations originating in the medial inferior temporal gyrus, extending through the lateral parietal-temporal-occipital gyrus, and eventually penetrating to the motor area and prefrontal gyrus with the illness duration increased (Fig. 5).

The hippocampus/parahippocampus is a significant region of interest in migraine research, as it is involved in pain processing, pain-related attention and memory, and stress response [31]. A growing number of studies have identified the structural, functional and metabolic abnormalities of the hippocampus/parahippocampus in both migraine patients and models [32–35]. Utilizing the VBM method, previous studies have demonstrated aberrant gray matter morphology in the hippocampus/parahippocampus in MwoA patients [35–37], as well as a more significant GMV increase in this region in patients with episodic migraine compared to those with chronic migraine [38]. Our current study added to this literature by demonstrating a significant GMV increase in the PHG.L in MwoA patients. Additionally, the subgroup analysis revealed GMV hypertrophy in PHG.L in patients with longer disease durations, as opposed to shorter ones. The correlation analysis found a significant, but not very strong correlation between gray matter changes and illness duration rather than the number and severity of headache attacks. These findings may suggest that migraine-related increases in parahippocampal volume are more likely to be a result of long illness duration or a greater number of episodes rather than a pre-existing vulnerability factor. This inference was confirmed in a small-sample longitudinal study [13], showing greater parahippocampus and temporoparietal gyrus alterations in individuals with a long disease duration and higher baseline disease activity at the four-year follow-up. Furthermore, research evidence from randomized controlled trials suggested that structural and functional alterations in the hippocampus/parahippocampus of MwoA were reversible [39–41]. For instance, Liu J. et al. [39] reported that some migraineurs exhibited significant clinical improvements and reduction in GMV of the abnormally enlarged amygdala/parahippocampus, indicating that hippocampal hypertrophy may be a state marker associated with prolonged duration or recurrence of migraine, rather than a permanent scar.

The SCN analysis was designed to explore the synchronous coordinated structure of the entire brain in gray matter morphology, as opposed to focusing on a particular region. This approach provides a valid method to investigate the zero time-lagged conjugating structures among the interconnected regions of the brain [31]. The results of the SCN analysis revealed synchronous changes in GMV among the parahippocampus and its neighboring regions, such as the hippocampus, amygdala, lingual gyrus, and cerebellum, rather than distant brain regions in MwoA patients. It suggested that the morphological aberrations in MwoA patients were highly concentrated, with the most pronounced synchronous alterations in the medial inferior temporal gyrus and cerebellum. These findings were highly concordant with the results of our latest coordinate-based mate analysis [8]. In this mate analysis, we enrolled 31 original studies and detected that MwoA patients had increased gray matter in the left amygdala, bilateral parahippocampus, right hippocampus, and left lingual gyrus. Furthermore, some other studies also found that MwoA patients manifested altered synchronization of functional activity in the medial temporal gyrus [42, 43] and that interventions such as transcutaneous auricular vagus nerve stimulation [44] and acupuncture [45] had significant modulating effects on these disturbed functional activity patterns of patients. This phenomenon suggested that there were consistent structural–functional variations in the medial inferior temporal gyrus in MwoA patients and that the cortical plasticity in the medial inferior temporal gyrus might be an important neuropathological feature of MwoA. Additionally, these regions, especially the parahippocampus, should be considered when developing neuromodulation methods for MwoA.

Another principal finding of the current study was that the parahippocampus might be a "driving core" of gray matter alterations in the MwoA. Namely, the hypertrophy of the parahippocampus preceded and predicted gray matter changes in other regions. It is well known that longitudinal study is a preferred approach for assessing morphological changes in the brain over time. However, acquiring data from the same subject over a prolonged period is often challenging. Therefore, the CaSCN method was developed as a surrogate measure of disease progression, incorporating information on the duration of the illness to produce a pseudo time series of disease progression and lifespan data [18]. The ability of the CaSCN method in capturing temporal variations of brain structure has been well validated [23, 46–48]. For example, Jiang et al. [23] found that patients with Schizophrenia had GMV atrophy originating in the thalamus and expanding to other regions, which is consistent with the findings of a longitudinal study [49]. Gray matter alterations in the hippocampus/parahippocampus of migraine patients have been widely recognized in cross-sectional studies [31]. Moreover, longitudinal studies [13, 50] demonstrated that the gray matter of the temporal, somatosensory, and prefrontal gyrus of migraineurs exhibited progressive alterations as the disease advances and that progressive gray matter damage in the prefrontal gyrus and temporal gyrus was more prominent in patients with worsening migraine compared to those with improving or stable disease. These findings, along with the results of our current study, confirmed the reliability of CaSCN-based investigation of gray matter morphology in MwoA patients and together revealed the time-related pattern of neural plasticity of migraine transiting from the initial phase to the chronic pain phase, which was manifested as the gradual infiltration from the medial inferior temporal gyrus to the lateral parietal-temporal-occipital gyrus and the motor cortex and prefrontal gyrus. As reported in the previous study [51, 52], pain is a complex and subjective experience that is shaped by evaluation and judgments about afferent sensory information in multiple brain regions, including the prefrontal gyrus, basal ganglia, sensorimotor cortex, and temporal-occipital cortex. Among these regions, the hippocampus, parahippocampus, and amygdala are mainly associated with the long-range memory of pain [53], the lateral parietal-temporal-occipital is involved in the integration of multisensory information during migraine attacks [54], while the prefrontal gyrus and motor cortex are largely involved in the cognitive, emotional, and motivational dimensions of pain [55]. Therefore, a plausible explanation regarding the current findings is that the long-term negative pain memories caused structural disruptions of the hippocampus/parahippocampus, interfered with the integration of multisensory information in the lateral parietal-temporal-occipital cortex and further affected the emotional and cognitive functions of the MwoA patients, resulting in the structural plasticity of the motor and prefrontal cortices.

Despite the meaningful findings in the current study, several methodological limitations should be stressed. Firstly, the CaSCN values were calculated based on the pseudo time series, implying only the extension of causal effects rather than directly reflecting the real progression of gray matter morphology in MwoA patients. Secondly, as the analysis of SCN and CaSCN are group-based, it is unavailable to obtain the subject-level values for all patients, thus impeding the exploration of their relationship with clinical measurements of migraine. Thirdly, the MwoA patients included in the study were concentrated between 20 and 30 years of age. Whether the current findings are applicable to patients in other age groups is yet to be verified. Finally, illness duration was used to describe the progression of MwoA, other clinical variables, such as illness severity, which may be associated with brain alterations, should also be considered in the future.

## Conclusion

In conclusion, our results indicated that gray matter structural alterations in the medial inferior temporal gyrus, especially the parahippocampus, were critical and initial pathological characteristics in MwoA patients. With the increase of illness duration, the GMV alterations of MwoA patients expanded from the medial inferior temporal gyrus and cerebellum to the lateral parietal-temporal-occipital cortex and further projected to the motor cortex and prefrontal gyrus, suggesting a parahippocampus-driven hierarchy alteration patterns of gray matter morphology in the progression of migraine. These findings provided further evidence for understanding the progressive gray matter morphological changes in migraine and may facilitate the development of neuromodulation methods targeting this procession.

## Data Availability

The main data supporting our findings can be found within the manuscript. Access to the raw data can be provided upon request.

## Abbreviations

MwoA: Migraine without aura
MRI: Magnetic resonance imaging
GC: Granger causality
CaSCN: Causal Structural Covariance Network
VBM: Voxel-Based Morphometry
GMV: Gray matter volume
HCs: Healthy controls
SCN: Structural Covariance Network
VAS: Visual Analogue Scale
MNI: Montreal Neurological Institute
TIV: Total intracranial volume
GRF: Gaussian Random Field
FDR: False discovery rate
ROI: Region-of-interest
PHG.L: Left parahippocampus
SMA: Supplementary motor area
AMY/aPHG: Amygdala/anterior parahippocampus
IOFG: Inferior orbitofrontal gyrus
